# Assessing preoperative plasma growth-differentiation factor-15 for prediction of acute kidney injury in patients undergoing cardiac surgery

**DOI:** 10.1186/s13054-017-1623-3

**Published:** 2017-03-17

**Authors:** Wei Zhang, Hai Chen Chu, Feng Xue

**Affiliations:** 1 Department of Nephrology, The Affiliated Hospital of Qingdao University, Qingdao, China; 2 Department of Anesthesiology, The Affiliated Hospital of Qingdao University, 16 Jiangsu Road, Qingdao, 266003 China

With great interest, we read the recent article published by Heringlake et al. [1] assessing whether preoperative plasma GDF-15 independently predicts postoperative AKI in patients undergoing elective cardiac surgery. Many factors in this study were done well. Other than strict inclusion and exclusion criteria of patients, the authors had also tried to control most of the known risk factors that can affect AKI. We thank the authors for their endeavor to validate that preoperative plasma GDF-15 independently predicts postoperative AKI in patients undergoing elective cardiac surgery. Nevertheless, other than the limitations described in the discussion, we note that several important issues of this study were not addressed.

First, preoperative aspirin therapy was not included in the data analysis. Actually, preoperative aspirin therapy is common among patients undergoing cardiac surgery for primary or secondary prevention of myocardial infarction, stroke, and death. Preoperative aspirin therapy in cardiac surgery has been associated with decreased overall morbidity, mortality, and cardiac surgery-associated acute kidney injury [2].

Second, patients receiving valvular heart surgery or coronary artery bypass graft surgery were included in the study. However, some patients may use contrast agents before surgeries. The preoperative contrast angiography or ventriculography is independently associated with increased risks of postoperative adverse renal events [3]. Therefore, contrast angiography or ventriculography should be included in the data analysis.

Third, it was unclear whether serum creatinine levels applied in the diagnosis of postoperative AKI had been adjusted based on the perioperative fluid balance. Moore et al. [4] showed that using Acute Kidney Injury Network criteria for diagnosis of AKI, if not adjusting serum creatinine levels for fluid balance, can underestimate the severity and incidence of AKI and can confuse the association of AKI with postoperative adverse outcomes.

Finally, when assessing the association of preoperative plasma GDF-15 with postoperative AKI in patients undergoing cardiac surgery, the available literature shows that postoperative complications including anemia, low cardiac output syndrome, hypoalbuminemia, and sepsis are independent risk factors of AKI after cardiac surgery [5]. We are concerned that not taking postoperative covariates into account would have biased the true effect of preoperative plasma GDF-15 on the occurrence of postoperative AKI in this study.

## Abbreviation

AKI: Acute kidney injury
