# Supplementary material for: Assessing preoperative plasma growth-differentiation factor-15 for prediction of acute kidney injury in patients undergoing cardiac surgery
Source: Crit Care. 2017 Mar 17;21:53. doi: 10.1186/s13054-017-1623-3 (PMC5356358; doi:10.1186/s13054-017-1623-3)
Supplement: Related texts

# Reply: Authors’ response

**Authors:** M. Heringlake, E. I. Charitos, K. Erber, A. E. Berggreen, H. Heinze, H. Paarmann

We thank Dr Zhang et al. for their interest in our work [1]. The authors raised concerns that our findings might be confounded by several variables not addressed specifically in our publication.

We are of course aware that every tool used for preoperative risk stratification must have relevant limitations, especially if aiming for the prediction of a multifactorial complication like cardiac surgery-associated acute kidney injury (CSA-AKI). However, we took the greatest care to take into account as many preoperative factors as possible.

Zhang et al. criticized that preoperative treatment with aspirin was not included in our analyses. However, the incidence of AKI in patients preoperatively treated with aspirin was not different (*p* = 0.720) from the AKI incidence in patients not being treated with aspirin. Consequently this variable was not entered into the multivariate analyses.

We are also aware of the association between immediate preoperative radio contrast medium exposure and postoperative AKI [6]. To omit this potential confounder we focused only on elective surgery, and excluded urgent patients from the analysis; at least in our institution, elective patients are rarely subjected to an immediate preoperative contrast agent exposure.

We admit that not taking into account the postoperative fluid balance may have lead to underestimation of AKI incidence and severity. Several studies, including that by Moore et al. [4] cited by Zhang et al., have shown that adjusting creatinine levels to fluid balance and body weight will increase the probability to detect clinically significant AKI. However, precise data on fluid balance were not available for all patients and thus we could not adjust postoperative creatinine levels accordingly. Nonetheless, the current definitions of AKI [7] do not urge making such adjustments; and thus our findings are clearly valid according to current AKI criteria (based on creatinine).

We of course agree with Zhang et al.’s comment “that not taking postoperative covariates into account would have biased the true effect of preoperative plasma GDF-15 on the occurrence of postoperative AKI”. However, as stated in our manuscript [1], a tool for preoperative risk stratification will hardly be capable to predict the unpredictable: bleeding anemia (due to poor surgery!), unexpected low cardiac output syndrome (e.g., due to inadequate cardioplegia), postoperative hypoalbuminemia (due to excessive need for crystalloid fluids), and sepsis.

Taken together the authors’ comments do not alter our statement that preoperative plasma GDF-15 is an independent predictor of postoperative AKI in patients undergoing elective cardiac surgery and improves the predictive ability of the Cleveland Clinic Acute Renal Failure score.
